# Impact of Physical Exercise on Substance Use Disorders: A Meta-Analysis

**DOI:** 10.1371/journal.pone.0110728

**Published:** 2014-10-16

**Authors:** Dongshi Wang, Yanqiu Wang, Yingying Wang, Rena Li, Chenglin Zhou

**Affiliations:** 1 Department of Sport Psychology, School of Kinesiology, Shanghai University of Sport, Shanghai, China; 2 Center for Hormone Advanced Science and Education, Roskamp Institute, Sarasota, Florida, United States of America; Georgia Regents University, United States of America

## Abstract

**Objective:**

The goal of this meta-analysis was to examine whether long-term physical exercise could be a potential effective treatment for substance use disorders (SUD).

**Methods:**

The PubMed, Web of Science, Elsevier, CNKI and China Info were searched for randomized controlled trials (RCT) studies in regards to the effects of physical exercise on SUD between the years 1990 and 2013. Four main outcome measures including abstinence rate, withdrawal symptoms, anxiety, and depression were evaluated.

**Results:**

Twenty-two studies were integrated in the meta-analysis. The results indicated that physical exercise can effectively increase the abstinence rate (OR = 1.69 (95% CI: 1.44, 1.99), z = 6.33, *p*<0.001), ease withdrawal symptoms (SMD = −1.24 (95% CI: −2.46, −0.02), z = −2, *p*<0.05), and reduce anxiety (SMD = −0.31 (95% CI: −0.45, −0.16), z  =  −4.12, *p*<0.001) and depression (SMD  =  −0.47 (95% CI: −0.80, −0.14), z = −2.76, *p*<0.01). The physical exercise can more ease the depression symptoms on alcohol and illicit drug abusers than nicotine abusers, and more improve the abstinence rate on illicit drug abusers than the others. Similar treatment effects were found in three categories: exercise intensity, types of exercise, and follow-up periods.

**Conclusions:**

The moderate and high-intensity aerobic exercises, designed according to the Guidelines of American College of Sports Medicine, and the mind-body exercises can be an effective and persistent treatment for those with SUD.

## Introduction

Substance abuse, such as alcohol, nicotine, and illicit drugs, is one of the largest public health issues in the world. Figures from the World Health Organization (WHO) show that 2.5 million people die from alcohol abuse each year, and at least 15.3 million people have substance use disorders (SUD). Moreover, 120 countries have reported cases of HIV infection in drug addicts [1]. Substance abuse also increases the risk of spreading HIV and is an overall detriment to society, family, and individuals [2]–[4]. Currently, one of the most commonly used treatments for substance addiction is drug replacement therapy, using substances such as methadone or buprenorphine. Both methadone and buprenorphine are long-acting opioid agonists and used to treat opioid addiction, such as heroin, by reducing and/or eliminating the use of substances, relieving craving behavior, suppressing abstinence symptoms, and decreasing substance abuse-associated infective diseases transmission [5], [6]. However, like all opioids, both methadone and buprenorphine have risk of addiction and often have potential of drug-drug interactions [7]. Opioid substitutes are also linked to diabetes, nicotine addiction, and premature death [8]–[10]. Therefore, there is a strong interest in finding alternative treatments for SUD.

In general, physical exercise is characterized as a planned, organized, and repeated body movement that aims to promote or maintain physical fitness [11]–[13]. The most common physical exercises include aerobics (brisk walking and running) and mind-body exercises (Tai Chi Quan, Qigong, and Yoga). Compared to methadone and buprenorphine drug-replacement therapies, physical exercise has been recognized as a potential add-on treatment for SUD. For example, studies showed that subjects with regular physical exercise showed lower rates of SUD compared to people with less exercise [14], and regular physical exercise in adolescence provided a preventive effect on alcohol and illicit drug use in adulthood [15]. Furthermore, exercise training caused a significant reduction in daily use and craving for cannabis in marijuana-dependent adults [16], and enhanced the healing effect on SUD [17]–[19]. The positive effects of physical exercise on SUD have also been confirmed in animal experiments. For example, wheel-running can ease withdrawal behavior in mice with morphine-addiction [20], while voluntary treadmill exercise [21], [22] and mandatory treadmill exercise [23] can reduce cocaine, morphine, nicotine, and alcohol intake in various mouse models [22], [24], [25].

However, some contradictory findings were reported, such as exercise providing no significant effects on substance abusers. For example, one study reported that a 3- week regiment of aerobic exercise and strength training failed to increase the abstinence rate of alcohol abusers [26], and another study found that a 10-week physical exercise program caused no change in the abstinence rate of smokers or relief for emotional symptoms related to smoking [27]. As there is no clear answer for these controversial findings, more comprehensive analyses of physical exercise, such as the intensity and duration of exercise applied, are needed.

In the past two years, a number of articles reviewed whether physical exercise could be considered as a potential method for treating SUD [13], [18], [28]–[35]. These articles included preclinical and clinical literature of physical exercise-induced protective effects on the different transitional phases of SUD. These include the initiation of drug use, the progression from use to addiction, the drug withdrawal and relapse period [29] in alcohol, nicotine and illicit drug use disorders [32], and the improvement of mood and overall life quality of those with SUD [13]. A few articles also reviewed the relationship between various type of physical exercises as promising complementary therapies for SUD [33], [34]. To further understand the relationship between physical exercise intervention and SUD, several statistical review articles have analyzed the effects of acute exercise on nicotine addiction. For example, Ussher *et al.* found that physical exercise can effectively intervene in symptoms related to smoking (RR: 0.97–4.96) using meta-analyses on thirteen [36] and fifteen [37] original research articles, while others reported similar results using analyses of individual participant data (IPD) or the systematic review method [38]. Together, these studies provide the support for using physical exercise as a treatment for SUD. However, there is a shortage of important evidence in previously published meta-analyses of physical exercise as treatments in SUD, such as the effect of mind-body exercise or chronic physical exercise on substance addiction with one or polydrugs, as well as a systematic evaluation of randomized controlled trials (RCT). A recent study reported that Yoga, a typical mind-body exercise, may improve mood status and quality of life for women undergoing detoxification for heroin dependence [39]. In addition, subjects who are addicted to more than one drug often develop more complicated symptoms related to the synergistic effect of drug-drug interaction on brain structures and functions [40]. It is known that acute exercise produces different effects on brain function, such as cognition, than long-term routing exercises, which can lead to improvement of object recognition memory and reduction of perceived stress [41]. Indeed, both acute and chronic aerobic exercises have been extensively used to treat SUD. The changes induced by acute exercise can be viewed as a transitory modulation of the arousal physiology [42], while effects of chronic physical exercise are generally explained by structural and durable changes in the organism, such as angiogenesis [43] and neurogenesis [44]. All of these may help to explain the contradictory findings on exercise intervention in SUD.

The aim of this meta-analysis is to verify the treatment effects of chronic physical exercise on various SUD by analyzing the current RCT studies. The abstinence rate, withdrawal symptoms, anxiety levels, and depression levels are included in this meta-analysis as outcomes of treatment. Furthermore, we also included the analyses of exercise intensities, exercise types, and lasting effects of physical exercise on SUD. Lastly, we performed sub-group analyses to provide details of potential optimal physical exercise therapies for specific drug addictions.

## Methods

This meta-analysis followed the PRISMA guidelines [45] for conducting and reporting systematic reviews.

### Search strategy

We conducted a search for relevant literature in the following electronic databases: PubMed, Web of Science, Elsevier, China National Knowledge Infrastructure (CNKI), and China Info. The key search words included exercise, physical activity, qigong, tai chi, yoga, heroin, morphine, opioid, opiate, cocaine, methadone, marijuana, cannabis, alcohol, drinker, cigarette, smoke, nicotine, drug abuse, drug dependence, and substance use. The search was limited to Chinese and English literature studying adults (≥18 years old) published from January 1990 to August 2013.

### Study selection and quality assessment

During reviewing relevant papers, data extraction and analysis complied in accordance to the following standards: (1) The selected papers were studying physical exercise intervention’s effect on drug abuse, excluding preventive studies. (2) All research use RCT. (3) Objects of the study were adults over 18 years old who were assessed as alcohol, nicotine, and illicit drug abusers through the DSM-III(R)/IV. (4) Excluding the studies on acute exercise, we selected results from chronic physical exercise experimental studies. (5) The primary outcome measures in the study included the rate of abstinence from drug addiction, withdrawal symptoms, the level of depression, and anxiety. (6) The baseline of the primary outcome measures in the study and descriptive statistical data after intervention must be obtainable.

The Delphi List Criteria was used to [46] assess the quality of each literature included in the meta-analysis. Our literature evaluation criteria included: randomness of grouping, concealment of treatment allocation, homogeneity of baseline data, clarity of various standards, viability of using the blind method for outcome measurement, assessment tools for the main outcome, and intent treatment analysis. In the current research, items 6 and 7 in the Delphi List are not integrated in the assessment. When measuring the effect of physical exercise on SUD, treatment providers need to guide and monitor patients to execute physical exercise intervention, making a blind method impossible.

### Data extraction and statistical analysis

We used an Excel spreadsheet to extract the data from the integrated literature. The data included information of participants, intervention of experimental and control groups, types of drug addiction, and primary outcome measurements. We conducted a meta-analysis through the meta package in R software (R 3.0.1 version) [47]. We used the odd ratio (OR) to assess the abstinence rate under two different conditions. Due to different follow-up intervals, we defined follow-up periods of 1–3 months, 4–7 months, and ≥8 months as short, middle, and long term, respectively. We evaluated the treatment effect by measuring abstinence rates at the end of physical exercise and throughout different follow-up periods, and then conducted a sub-group analysis based on different follow-up phases. We also employed the standardized mean difference (SMD) to assess withdrawal symptoms, depression, and anxiety after physical exercise intervention. The confidence interval was set at 95%. A *p* value less than 0.05 is referred to the level of statistical significance. We used *Q*-test and *I* ^2^-test to assess heterogeneity. If the *p* value in *Q*-test was less than 0.05 or if the *I* ^2^ index in *I* ^2^-test was more than 50%, the data was judged to achieve heterogeneity [48], [49]. If there was heterogeneity, the random effects model was chosen in the meta-analysis; otherwise, the fixed effects model was used. Given the heterogeneity in the study, we rendered sensitivity analysis. We employed the funnel plot visual, Egger’s test, and false safe number (*N_fs0.05_*) to assess publication bias in the meta-analysis including more than ten papers [50]. In addition, we performed sub-group analysis according to certain characteristics of participants in the studies (physical exercise intensity, physical exercise type, addictive type, and follow-up period).

Authors WYQ and WYY completed the screening of the literature, data extraction, quality analysis, and statistical analysis process independently. Meetings were held regularly to minimize the risk of error in each link. In case of conflicts between two authors in any process, the final decision was made by another author.

## Results

### Included trials

**Figure 1:**
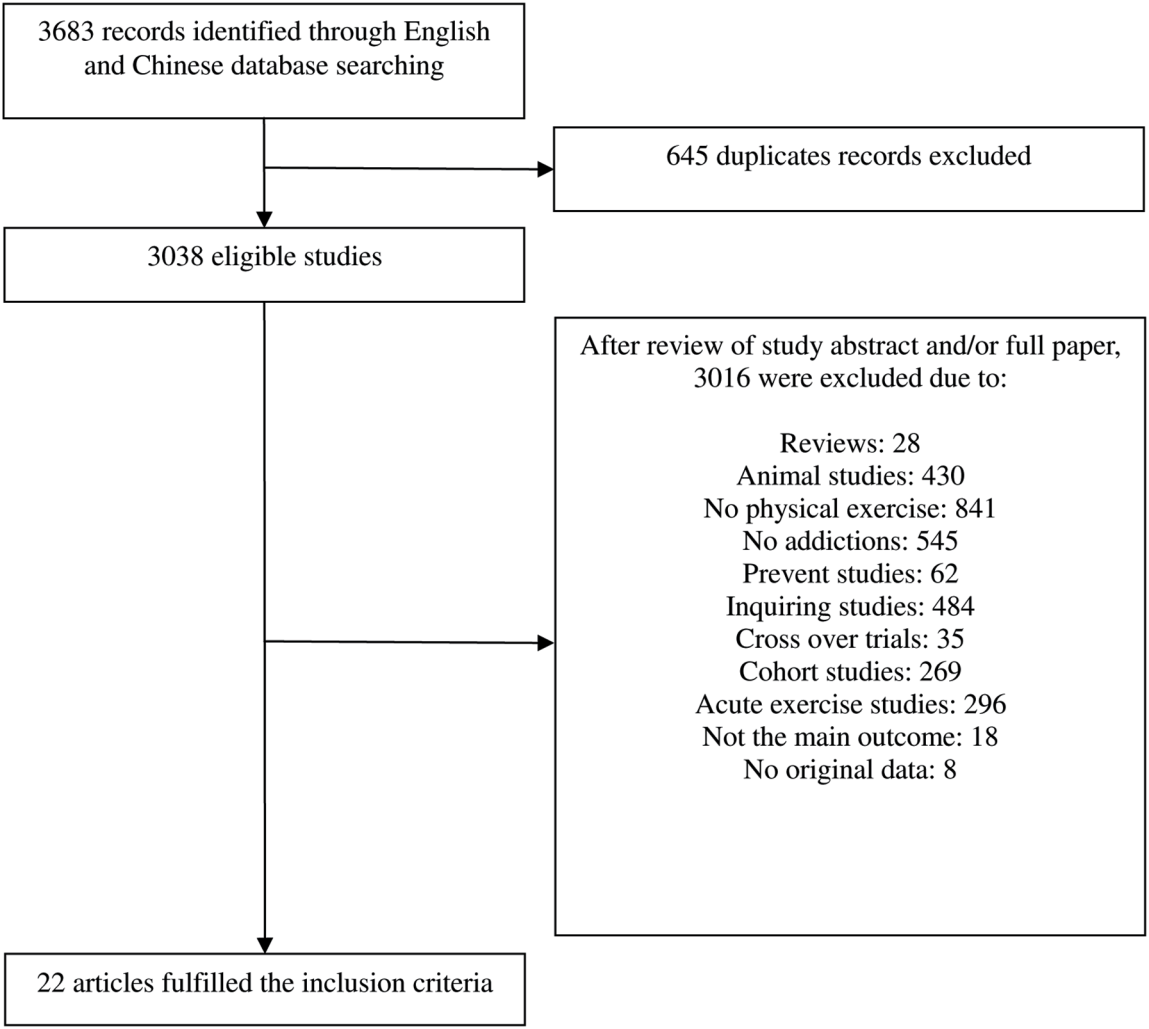
Flow chart of trials used in the study.

Based on the selection criteria, we derived 22 out of 3683 studies for meta-analysis. The flow diagram of identification, screening and the inclusion of studies was shown (see Figure 1). One doctoral dissertation was selected [26], and the original data of one study was provided by contacting the author [51]. Five papers from 22 studies investigated the treatment effects of physical exercise on illicit drug abusers [39], [51]–[54], eleven on nicotine abusers [27], [55]–[64], three on alcohol abusers [26], [65], [66], and four on polydrug abusers of alcohol, nicotine, and illicit drugs [26], [67]–[69]. Table 1 shows the specific characteristics of the included studies. Among all the studies, abstinence rate was regarded as the primary outcome measure with four reporting only the abstinence rate or abstinence population after physical exercise intervention [52], [53], [64], [67]. Other papers reported abstinence conditions immediately after intervention and in different follow-up periods. Moreover, five papers reported withdrawal symptoms [51]–[54], [57], seven papers described changes in anxiety levels before and after physical exercise intervention [26], [51], [53], [57], [63], [66], [69], and nine present variety in depression levels before and after physical exercise intervention [26], [27], [39], [53], [54], [63], [65], [66], [69].

**Table 1:** Characteristics of included studies.

Article	Substance	Group	N	Age	Gender	Race	Physical Exercise					Outcomemeasure
							Type	Intensity	Duration	Frequency	Each time	
Burling (1992)[67]	Illicit drug,alcohol	Exp	34	38.8	2.9%F	50%Black;41%Caucasian	Softball	NR	30 day	1/Wks	NR	Abstinent rate^a^
		Ctrl	61	NR	NR	NR	No Exercise	NR	30 day	NR	NR	
Marcus (1995)[55]	Nicotine	Exp	10	39	F	NR	Walking,rowing, cycleergometry	70–85%HR max	12 week	3/Wks	30–45 min	Abstinent rate^a^
		Ctrl	10	36	F	NR	Educationmeeting	NR	12 week	1/Wks	30–45 min	
Donaghy (1997)[26]	Alcoholic,	Exp	80	41.3	25%F	NR	Aerobicexercise,strengthening	NR	3 week	3/Wks	30 min	Abstinent rate^a^; BDI; STAI(state)
		Ctrl	78	41.7	25%F	NR	Breathing	NR	3 week	3/Wks	30 min	
Martin (1997)[68]	Nicotine,alcohol,illicit drug	Exp	72	40.5	43%F	90.4%Caucasian	Walking	Moderate	8 week	3/Wks	15–45 min	Abstinent rate^a^
		Ctrl	70	41.5	46%F	95.7%Caucasian	Standardtreatment	NR	8 week	NR	60–70 min	
Marcus (1999)[56]	Nicotine	Exp	134	40.7	F	NR	Walking,rowing, cycleergometry	69–85%HR max	12 week	3/Wks	40 min	Abstinent rate^a^
		Ctrl	147	39.7	F	NR	Educationmeeting	NR	12 week	1/Wks	30 min	
Huang (2000a)[52]	Heroin	Exp	60	NR	30%F	Asia/Chinese	Jogging	40–70%VO_2 max_	10 day	3/Wks	40–60 min	Withdrawal symptoms^c^; Abstinent rate^a^
		Ctrl	60	NR	33%F	Asia/Chinese	Daily life	7–8 Borg^b^	10 day	NR	NR	
Huang (2000b)[53]	Heroin	Exp	60	27.0	30%F	Asia/Chinese	Brisk walking	50–60%VO_2 max_	6 month	3/Wks	40–60 min	Withdrawal symptoms^c^; Abstinent rate^a^; SAS; SDS
		Ctrl	50	28.2	27%F	Asia/Chinese	Daily life	9–10 Borg^c^	6 month	NR	NR	
Li (2002) [51]	Heroin	Exp	34	33.3	M	Asia/Chinese	Qi Gong	NR	10 day	4–5/D	25–30 min	Withdrawal symptoms^c^; HAS
		Ctrl	26	31.7	M	Asia/Chinese	NR	NR	10 day	NR	NR	
Ussher (2003)[57]	Nicotine	Exp	154	41.5	53%F	87.9%Caucasian	‘Life-style’or morestructuredexercise	40% HRmax	6 week	5/Wks	5–30 min	Withdrawal symptoms^d^; Abstinent rate^a^ MPSS
		Ctrl	145	44.4	53%F	87.9%Caucasian	Healtheducation	NR	6 week	1/Wks	5–30 min	
Marcus (2005)[58]	Nicotine	Exp	109	42.5	F	82.5%Caucasian; 6.9%Black	Aerobictraining	50–69%HR max	8 week	4/Wks	30–45 min	Abstinent rate^a^
		Ctrl	108	43.0	F	82.5%Caucasian; 6.9%Black	Healtheducation	NR	8 week	NR	30–45 min	
Vedamurthachar (2006) [65]	Alcohol	Exp	30	35.6	M	NR	Sudarshana Kriya Yoga	NR	2 week	NR	60 min	BDI
		Ctrl	30	37.7	M	NR	No intervention	NR	2 week	NR	NR	
Sareen (2007)[66]	Alcohol	Exp	26	50	14%F	NR	Iyengar Yoga	NR	12 week	2/Wks	60 min	POMS
		Ctrl	26	50	14%F	NR	Usual care	NR	12 week	NR	NR	
Prapavessis(2007) [59]	Nicotine	Exp	76	37.9	F	NR	Walking, rowing,cycle ergometry	60–75%HR max	12 week	3/Wks	45 min	Abstinent rate^a^
		Ctrl	66	38.2	NR	NR	Cognitivebehavior therapy	NR	12 week	NR	NR	
Ussher (2007)[60]	Nicotine	Exp	154	41.5	53%F	87.9%Caucasian	‘Life-style’ ormore structuredexercise	40% HRmax	6 week	5/Wks	5–30 min	Abstinent rate^a^
		Ctrl	145	44.4	53%F	87.9%Caucasian	Health education	NR	6 week	1/Wks	5–30 min	
Kinnunen (2008)[61]	Nicotine	Exp	92	38.3	F	81.5%Caucasian	Treadmill	60–80%HR max	19 week	1–2/Wks	30 min	Abstinent rate^a^
		Ctrl	34	39.9	F	75.8%Caucasian	Standard treatment	NR	19 week	NR	30 min	
Vickers (2009)[27]	Nicotine	Exp	30	41.8	F	Caucasian	Exercise	NR	10 week	5/Wks	30 min	Abstinent rate^a^; HRSD
		Ctrl	30	40.9	F	97%Caucasian	Health counseling	NR	10 week	1/Wks	30 min	
Williams (2010)[62]	Nicotine	Exp	29	41.5	F	83.3%Caucasian	Treadmill	70% HRmax	8 week	3/Wks	50 min	Abstinent rate^a^
		Ctrl	30	43.3	F	86.7%Caucasian	Wellness videos	NR	8 week	3/Wks	30 min	
Bock (2012)[63]	Nicotine	Exp	32	43.8	F	88%Caucasian	Yoga	NR	8 week	2/Wks	60 min	Abstinent rate^a^; STAIT; CESD
		Ctrl	23	48.1	F	74%Caucasian	Wellness sessions	NR	8 week	NR	NR	
Whiteley (2012)[64]	Nicotine	Exp	166	44.1	F	NR	Aerobic &resistance training	77–85%HR max	12 week	1/Wks	40–60 min	Abstinent rate^a^
		Ctrl	164	42.9	F	NR	Wellness session	NR	12 week	NR	NR	
Li (2013) [54]	Heroin	Exp	17	30.3	F	Asia/Chinese	Tai Chi	NR	150 day	1–2/D	60 min	Withdrawal symptoms^c^; HRSD
		Ctrl	16	29.6	F	Asia/Chinese	Daily life	NR	150 day	NR	NR	
Smelson (2013)[69]	Cocaine, alcohol	Exp	51	30.6	4%F	60%Caucasian; 35%Black	Qi Gong	NR	14 day	2–3/Wks	15 min	SAIS(state); BDI
		Ctrl	50	40.4	4%F	60%Caucasian; 35%Black	Sham Qi Gong	NR	14 day	NR	15 min	
Zhuang (2013)[39]	Heroin	Exp	37	29.1	F	Asia/Chinese	Yoga	NR	6 month	5/Wks	50 min	POMS(depression)
		Ctrl	38	27.8	F	Asia/Chinese	Hospital routinecare	NR	6 month	NR	NR	

Exp: Experimental; Ctrl: Control; F: Female subjects; NR: No Reported; HR: Heart Rate; VO_2 max_: maximal oxygen consumption; BDI: Beck Depression Inventory; SAIS: State-Trait Anxiety Inventory-State; TAIS: Trait Anxiety Inventory-State; SAS: Self-Rating Anxiety Scale; SDS: Self-rating depression scale; HAS: Hamilton Anxiety Scores; MPSS: Mood and Physical Symptoms Scale-anxiety; HRSD: Hamilton Rating Scale for Depression; CESD: Center for Epidemiologic Studies Depression Scale; POMS: Profile of Mood States.

a Continual abstinence;

b Borg index;

c Rating scale of heroin withdrawal symptoms;

d Tobacco withdrawal symptoms.

### Methodological Quality of Included Studies

Delphi List Criteria, which assesses the quality of RCT methodology [46], was used for quality assessment of all studies. Scores of quality ranged from 4 to 7 (see Table 2), which indicated that the bias of the study was relatively low. Some authors did not clearly report the experimental information such as whether the assessment of outcome adopted the blind method, or if sufficient concealment was made in the allocation of participants.

**Table 2:** Assessment of Methodological Quality of Included Studies.

	Randomization^a^	Similar atbaseline^b^	Criteriaspecified^c^	Assessorblinded^d^	Allocationconcealment^e^	Variabilityoutcome^f^	ITA^g^	Totalscore
Burling (1992)[67]	Unknown	YES	YES	Unknown	YES	YES	YES	5
Marcus (1995)[55]	YES	YES	YES	Unknown	Unknown	YES	YES	5
Donaghy (1997)[26]	YES	YES	YES	Unknown	Unknown	YES	YES	5
Martin (1997)[68]	YES	YES	YES	Unknown	YES	YES	NO	5
Marcus (1999)[56]	YES	YES	YES	YES	Unknown	YES	YES	6
Huang (2000a)[52]	YES	YES	YES	YES	Unknown	YES	NO	5
Huang (2000b)[53]	YES	YES	YES	YES	Unknown	YES	YES	6
Li (2002) [51]	YES	YES	YES	Unknown	Unknown	YES	YES	5
Ussher (2003)[57]	YES	YES	YES	Unknown	Unknown	YES	YES	5
Marcus (2005)[58]	YES	YES	YES	Unknown	Unknown	YES	YES	5
Char (2006)[65]	YES	YES	YES	YES	Unknown	YES	YES	6
Sareen (2007) [66]	YES	YES	YES	NO	Unknown	YES	YES	5
Prapavessis (2007)[59]	YES	YES	YES	YES	Unknown	YES	YES	6
Ussher (2007)[60]	YES	YES	YES	YES	YES	YES	YES	7
Kinnunen (2008)[61]	YES	YES	YES	Unknown	YES	YES	NO	5
Vickers (2009)[27]	YES	YES	YES	Unknown	YES	YES	NO	5
Williams (2010)[62]	YES	YES	YES	Unknown	Unknown	YES	NO	4
Bock (2012)[63]	YES	YES	YES	Unknown	Unknown	YES	YES	5
Whiteley (2012)[64]	YES	YES	YES	Unknown	YES	YES	YES	6
Li (2013)[54]	YES	YES	YES	Unknown	YES	YES	NO	5
Smelson (2013)[69]	YES	YES	YES	Unknown	Unknown	YES	NO	4
Zhuang (2013)[39]	YES	YES	YES	Unknown	YES	YES	NO	5

a Was randomization performed?

b Were the groups similar at baseline regarding important prognostic indicators?

c Were the eligibility criteria specified?

d Was the outcome assessor blinded?

e Was allocation concealment adequate?

f Were point estimates and measures of variability presented for the primary outcome measures?

g Did the analysis include an intention to treat analysis?

### Effect of physical exercise on abstinence rate

**Figure 2:**
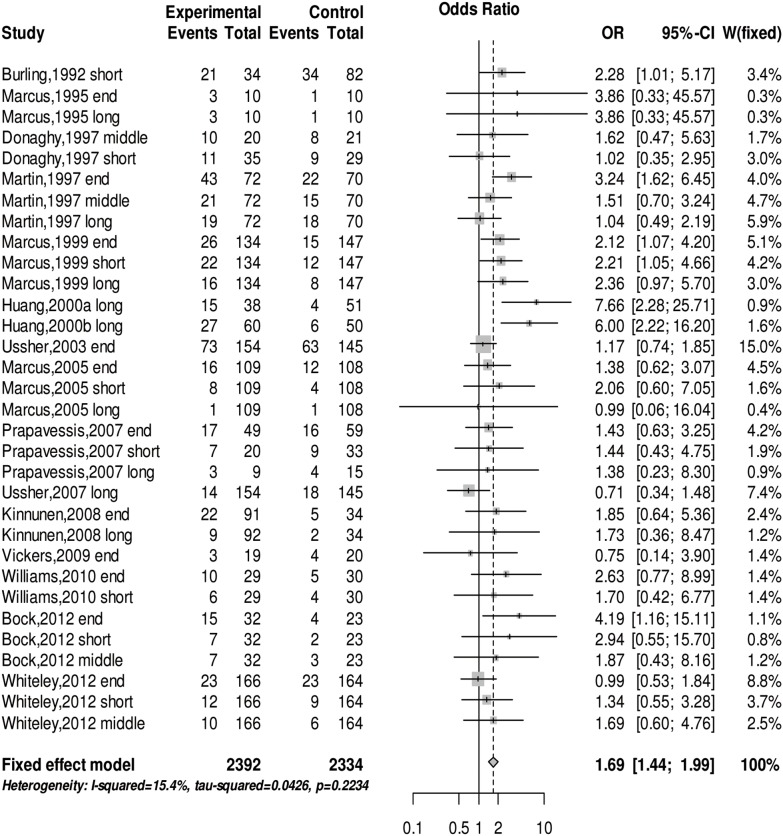
The forest plot about the effect of physical exercise on abstinence rate. The abstinence rate of past physical exercise treat, and differences follow-up periods were used odds ratio analysis.

We conducted a meta-analysis of the abstinence rate for SUD at the end of the physical exercise treatment, the short-term (≤3 months), middle-term (4–7 months) and long-term (≥8 months) follow-up periods after the intervention. The *Q* test (*Q*(31)  = 36.64, *p* = 0.22) and *I^2^* test (*I^2^* = 15.4%) revealed no heterogeneity in any of the studies. The meta-analysis of the fixed effects model shows that physical exercise can significantly increase the abstinence rate in subjects with SUD (OR = 1.69 (95% CI: 1.44, 1.99), z = 6.33, *p*<0.001) (see Figure 2). There was no evidence of publication bias upon using Egger’s test (*z* = 1.02, *p* = 0.31) and a false safe number (*N_fs0.05_* = 351). Table 3 shows the result of sub-group analysis indicating the effect of physical exercise intervention on the abstinence rate in various follow-up periods having no significant difference. The effect of physical exercise intensity and type of physical exercise on abstinence rate is not significantly different. However, there is strong evidence indicating the special effect of physical exercise on various addictive drugs. The treatment effect of physical exercise on drug abusers is better than its effect on alcohol and nicotine abusers (see Table 3).

**Table 3:** Sub-group analysis results.

		Sample size		N ofstudies	Meta-analytic effect size			Heterogeneity	
		(Exp/Ctrl)			SMD/OR(95%-CI)	*Q(d.f.)*	*p*	*I^2^*	*Q*
**Abstinence rate**									
Intensity type									
	low		96/69	3	2.96(1.29,6.83)	1.91(2)	0.3841	0%	0.66
	moderate		1193/1244	19	1.62(1.32,1.98)			36.8%	28.49
	high		1103/1021	10	1.71(1.28,2.29)			0%	5.47
Physical exercise type									
	aerobic Ex		2296/2265	29	1.65(1.40,1.95)	1.82(1)	0.177	17.9%	34.10
	mind-body Ex		96/69	3	2.96(1.29,6.83)			0%	0.66
Follow-up period									
	end		865/810	11	1.60(1.26,2.02)	0.57(3)	0.9027	23.8%	13.12
	short-term		559/616	8	1.79(1.25,2.56)			0%	2.65
	middle-term		290/278	4	1.62(0.96,2.71)			0%	0.07
	long-term		678/630	9	1.84(1.30,2.59)			60.9%	20.45
Addict type									
	alcohol		271/260	5	1.65(1.14,2.39)	11.51(2)	0.0032**	33.1%	5.98
	illicit drug		132/183	3	4.13(2.39,7.14)			43.9%	3.56
	nicotine		1989/1891	24	1.51(1.24,1.83)			0%	16.40
**Withdrawal symptoms**									
Physical exercise type									
	aerobic Ex		274/255	3	−1.67(−3.51, 0.17)	0.71 (1)	0.399	98.5%	2.59
	mind-body Ex		51/42	2	−0.61(−2.25,1.03)			92.6%	1.30
**Anxiety**									
Addict type									
	alcohol		61/55	2	−0.21(−0.58, 0.16)	1.03(2)	0.5975	37.9%	1.61
	illicit drug		145/126	3	−0.40(−0.64, −0.16)			0%	1.41
	nicotine		186/168	2	−0.26(−0.47, −0.05)			0%	0.13
Physical exercise type									
	aerobic Ex		249/224	3	−0.29(−0.47, −0.11)	0.06(1)	0.8065	21.1%	2.54
	mind-body Ex		143/125	4	−0.33(−0.57, −0.09)			0%	1.57
**Depression**									
Physical exercise type									
	aerobic Ex		132/117	3	−0.43(−0.84, −0.03)	0.04(1)	0.838	59.4%	4.93
	mind-body Ex		186/175	6	−0.50(−1.00, −0.01)			81.5%	27.03
Addict type									
	alcohol		91/85	3	−0.77(−1.73,0.19)	8.21(2)	0.0165*	89.2%	18.52
	illicit drug		165/154	4	−0.51(−0.73, −0.28)			0%	2.81
	nicotine		62/53	2	0.10(−0.27, 0.46)			0%	0.01

Exp: Experimental; Ctrl: Control; Ex: Exercise; SMD: Standardized Mean Difference; OR: Odds Ratio; CI: Confidence Interval.

*p<0.05, **p<0.01

### Effect of physical exercise on withdrawal symptoms

**Figure 3:**
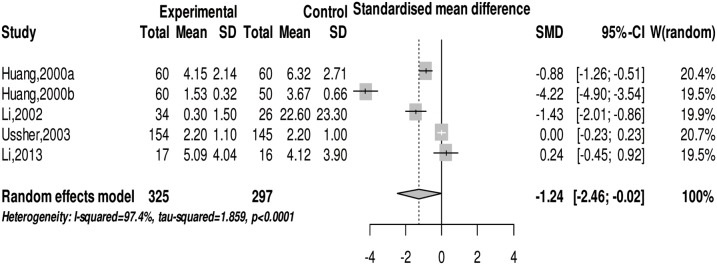
The forest plot about the effect of physical exercise on withdrawal symptoms.

We conducted a meta-analysis on withdrawal symptoms in drug abusers after physical exercise intervention. The *Q* test (*Q*(4) = 151.4, *p*<0.001) and *I^2^* test (*I^2^* = 97.4%) showed heterogeneity in the included studies. We chose the random effects model in meta-analysis and the result indicates that exercise can significantly ease withdrawal symptoms in subjects with SUD (SMD = −1.24 (95% CI: −2.46, −0.02), z = −2.00, *p*<0.05) (see Figure 3). The sub-group analysis finds that different types of physical exercise affect withdrawal symptoms of SUD differently (see Table 3).

### Effect of physical exercise on anxiety levels in subjects with SUD

**Figure 4:**
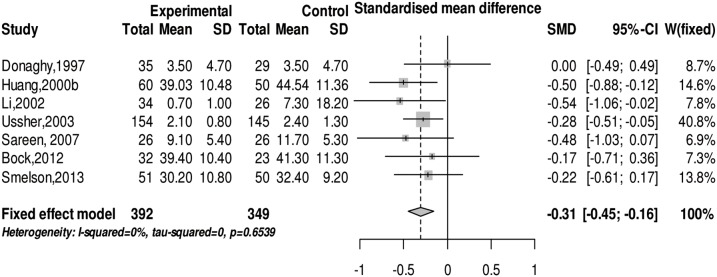
The forest plot about the effect of physical exercise on anxiety status.

We employed a meta-analysis on anxiety levels in subjects with SUD after physical exercise intervention. No evidence of heterogeneity was found based on the result of the *Q* test (*Q*(6) = 4.17, *p* = 0.65) and *I^2^* test (*I^2^* = 0%). The meta-analysis of the fixed effects model showed that physical exercise can significantly attenuate anxiety symptoms in subjects with SUD (SMD = −0.31 (95% CI: −0.45, −0.16), z = −4.11, *p*<0.001) (see Figure 4). The sub-group analysis finds that different types of physical exercise do not have significantly different effects on anxiety symptoms of addicts, and physical exercise does not differently influence the anxiety symptoms of all kinds of SUD (see Table 3).

### Effect of physical exercise on depression levels in subjects with SUD

**Figure 5:**
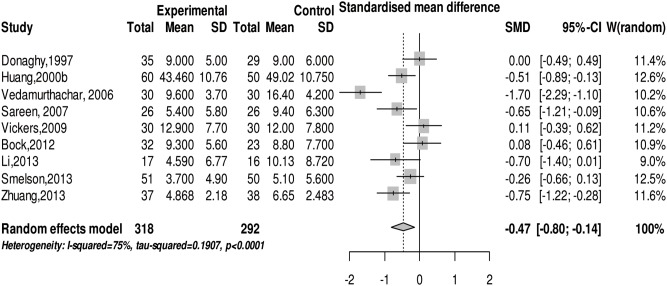
The forest plot about the effect of physical exercise on depression status.

We conducted a meta-analysis on the depression level in SUD after physical exercise intervention. There was moderate heterogeneity exhibited in the studies demonstrated by the *Q* test (*Q*(8) = 31.99, *p*<0.001) and *I^2^* test (*I^2^* = 75%). The random effects model meta-analysis showed that physical exercise can significantly relieve depression symptoms in SUD (SMD = −0.47 (95% CI: −0.80, −0.14), z = −2.76, *p*<0.01) (see Figure 5). The sub-group analysis indicated that the effect of physical exercise on the depression symptoms of SUD is not significantly different. However, physical exercise does have a significant relief effect on depression symptoms in alcohol and illicit drug abusers (see Table 3).

## Discussion

### Summary of main results

Based on our knowledge, our statistical review of physical exercise in SUD is the first meta-analysis article on the treatment effects of chronic physical exercise on alcohol, nicotine, and illicit drugs in human studies. Our meta-analysis assesses the effects of various forms of physical exercises on SUD in the following four aspects: abstinence rate, withdrawal symptoms, anxiety, and depression level. Compared to the treatment effects on alcohol and nicotine abusers, chronic physical exercises can better increase the abstinence rate in illicit drugs abusers. Meanwhile, physical exercise (aerobic exercise and mind-body exercise) can effectively attenuate withdrawal symptoms and ease anxiety symptoms in alcohol, nicotine and illicit drug addictions, while physical exercise-induced improvement of depression symptoms was only observed in illicit drug abusers. Furthermore, there are no significant differences in exercise-induced treatment on SUD between exercise types (aerobics vs. mind-body exercise), nor among different levels of physical exercise intensities (low, moderate, and high intensity). Overall, physical exercise can effectively assist abusers with withdrawing from addictive drugs. The result of this meta-analysis is not only consistent with the previous meta-analysis results from a single acute physical exercise treatment for SUD [38], [70], but it also complies with the result of several review articles [18], [29], [32].

#### Specificity of physical exercise on different addictive drugs

There are three types of addictive substances included in this analysis. They are alcohol, nicotine, and illicit drugs that induce strong dependence in human beings and could result in abuse. Since the different addictive mechanisms of these three substances may be involved, we performed sub-group analyses on each addictive substance independently. Indeed, we found that exercise affects the abstinence rate of illicit drugs more than that of alcohol and nicotine abuse. The possible cause for this result may be the difference in addictive mechanisms for each of the three addictive substances. For example, opioid drugs (morphine, heroin, etc.) take effect through β-endorphin neurotransmitters activating the µ and δ opium receptors [71]. Alcohol takes effect through promoting the reaction of the GABAA receptor [72] and increasing stimulation to the dopamine and opiate receptor [73], [74]. Nicotine takes effect through acetylcholine neurotransmitters activating the α2β4nACh receptor [75]–[77].

#### Physical exercise intensity for effectively treating drug abuse

The sub-group analysis indicated no significant difference in the abstinence rate among low-, moderate-, and high-intensity long-term physical exercise. It is mean that all three exercise intensities induce similar levels of abstinence rate reduction in alcohol, nicotine and illicit drug abusers. Correspondingly, non-RCT studies presented that long-term treadmill exercise at moderate-intensity (55–69% HR max) can effectively ease the craving for cannabis [16] and increase abstinence rate of other drugs in SUD [17]. And non-RCT studies also show that high- and moderate-intensity long-term physical exercise can significantly treat alcohol abuse. A pilot study on the treatment effects of aerobics on alcohol abusers [78] reported that 35 minutes of moderate- (HR = 50–60% VO2max) to high-intensity (HR = 80–90% VO2max) physical exercise for six weeks, can significantly reduce depression symptoms in alcohol abusers. Another cross-over design study showed that moderate-intensity fast walking can significantly enhance self-management techniques in alcohol abusers [79], [80]. The results of the above-mentioned study further proved the findings of our meta-analysis. While the mechanisms underlying these results remain unclear, moderate- and high-intensity aerobic exercises have significant effects on SUD. Mind-body exercises are included as low-intensity physical exercise in this meta-analysis. Due to the different nature of mind-body exercises compared to classical running and walking, we have included the analyses of mind-body exercises in SUD independently in the sections below.

#### Persistency of treatment effects of physical exercise on SUD

To investigate the long lasting effects of exercise on SUD, we conducted a sub-group analysis to evaluate the treatment effects of physical exercise in different follow-up periods. As shown in Table 3, there is no significant difference in exercise-induced reduction of the abstinence rate among the different follow-up periods,suggesting that physical exercise leaves a long lasting treatment effect on SUD. The results of two meta-analyses [36], [37] studies have also shown obvious treatment effects in 3 and 12 months follow-up periods. These results are likely due to the long lasting effect of physical exercise on changes in brain structure and function. For instance,studies showed that physical exercise can regulate the gene transcription of endogenous opium brain-derived neurotrophic factor (BDNF) by activating the cyclic AMP response element-binding (CREB) protein [81] and synaptic plasticity [82], [83], which is critical for rehabilitation for patients with SUD via promoting repair of drug-induced neuronal damage [84] and improving corresponding brain functions [85]. This neuronal structural change induced by exercises might contribute a long lasting effect on SUD.

#### Different effects of various forms of physical exercises

In addition to aerobic exercises, mind-body exercises are also acceptable and easy to apply in treatment for drug abuse. The sub-group analysis showed that mind-body exercises and aerobic exercises induce similar beneficial effects on the abstinence rate, withdrawal symptoms, anxiety, and depression levels in subjects with SUD (see Table 3). The outcome from our analysis is consistent with previously published literatures, suggesting that mind-body exercises can also effectively facilitate the treatment of drug addiction [35].

In terms of exercise intensity, some studies have shown that the intensity of Tai Chi Quan is about 52–63% HR max [86], which is similar to low- or moderate-intensity aerobic exercises [87]–[89]. Meanwhile, Qigong [90] and Yoga [91], [92] are also regarded as low- and moderate-intensity physical exercises. Therefore, both mind-body exercise and aerobic exercise show similar beneficial effects on drug addiction when administered at low- or moderate-intensities as shown in Table 3. In addition to exercise intensity, the particular characteristics of mind-body exercise, namely meditation and breathing exercises, may be the key reasons for producing significant exercise effectiveness [93], [94].

#### Physical exercise inducing emotional improvement in SUD

The meta-analysis indicates that physical exercise can effectively ease anxiety and depression symptoms in subjects with SUD. The sub-group analysis shows that physical exercises reduce anxiety symptoms in nicotine, alcohol and illicit drug abusers, while significant exercise-induced improvement on depression symptoms was only found in alcohol and illicit drug abusers (see Table 3). Our findings are consistent with previously published review articles which also found that physical exercise can effectively ease anxiety symptoms in subjects with SUD [29], [32], [95].

However, we also included three studies that reported that physical exercise did not reduce depression symptoms in nicotine and alcohol abusers [27], [63]. As the number of the RCT studies included in our meta-analysis is limited, it is insufficient to make a valid conclusion of whether exercise reduces depression symptoms in subjects with SUD. However, some review articles claim that physical exercise could attenuate depression symptoms in alcohol and nicotine abusers based on non-RCT studies [32], [70]. Hence, subsequent studies are needed for further evidence of the effect of exercise on altering depression symptoms in SUD.

### Limitations

There are some limitations that need to be considered in the current meta-analysis: (1) Addictive drugs involved in the studies often include participants with polydrug use disorders. Therefore, the specific treatment of exercise on a single drug abuse is difficult to analyze. Because there are insufficient RCT studies on exercise as an intervention for alcohol and drug abuse, the results in the present meta-analysis study may need further investigation. (2) Due to the limitation of available studies, only published RCT studies from 1990 to 2013 were included. There were no unpublished papers and dissertations found. These limitations in the literature collection may cause publishing bias. (3) Some papers lack clear explanations on the study information. Such limitation affected the quality of study and the assessment of clinic-relevant data. (4) In the research, some studies comprised of mostly female participants, whereas male participants accounted for only a small part. We were unable to evaluate the differences in gender through a statistical method due to difficulty in obtaining individual data. Accordingly, compared with males, female participants have more difficulty in giving up drug addiction [95]. (5) The main outcome indices in meta-analysis include abstinence rate, withdrawal symptoms, anxiety, and depression levels; the assessment tools in the literature also varied. (6) The included studies have some risk of bias. Lacking of assessor blinding and allocation concealment was the most frequent shortcoming of these studies (See Table 2). Other limitations included the small sample size of two studies [54], [55]. Although we adopted some methods (e.g., continuous variable adopts standardization mean difference method) for the assessment, certain biases listed above still exist.

## Conclusions

The current meta-analysis provides strong evidence that physical exercise can be an effective adjunct treatment method for abstinence from alcohol, nicotine, and illicit drugs in abusers. Physical exercise not only increases the abstinence rate in subjects with SUD, but also eases withdrawal symptoms, anxiety, and depression symptoms. The treatment effects of physical exercise in these four aspects verify that physical exercise guidance by the American College of Sports Medicine (ACSM) [12] is an effective means for drug abstinence. Additionally, mind-body exercises (including Tai Chi Quan, Qigong, and Yoga) have similar treatment effects as to aerobic exercise. Although physical exercise has been proven effective in facilitating drug abstinence, its effects on alcohol, nicotine and illicit drug abusers are different. From the results of the meta-analysis, the effects of physical exercise on illicit drugs abusers are significantly greater compared to the others. Given the limitation of materials, these issues require further investigation.

## Supporting Information

Checklist S1 **PRISMA Checklist.**
(DOC)

## References

[1] WHO (2013) Management of substance abuse. World Health Organization.

[2] CNNCC (2013) Annual Report on Drug Control in China 2013. Beijing: Ministry of Public Security.

[3] UNODC (2012) World Drug Report 2012. United Nations Publication.

[4] Deng Q, Tang Q, Schottenfeld RS, Hao W, Chawarski MC (2012) Drug use in rural China: a preliminary investigation in Hunan Province. Addiction 107: 610–613. 21906200 10.1111/j.1360-0443.2011.03648.x PMC4596718

[5] Joseph H, Stancliff S, Langrod J (2000) Methadone Maintenance Treatment (MMT):A Review of Historical and Clinical Issues. The Mount Sinai Journal of Medicine 67: 347–364. 11064485

[6] Mattick RP, Kimber J, Breen C, Davoli M (2014) Buprenorphine maintenance versus placebo or methadone maintenance for opioid dependence. Cochrane database of systematic reviews.

[7] Fischer B, Rehm J, Rgen U, Kim G, Kirst M (2004) Eyes wide shut?–A conceptual and empirical critique of methadone maintenance treatment. European addiction research 11: 1–14. 10.1159/000081410 15608466

[8] Maruyama A, Macdonald S, Borycki E, Zhao J (2013) Hypertension, chronic obstructive pulmonary disease, diabetes and depression among older methadone maintenance patients in British Columbia. Drug and alcohol review 32: 412–418. 23480234 10.1111/dar.12031

[9] Fareed A, Casarella J, Amar R, Vayalapalli S, Drexler K (2009) Benefits of retention in methadone maintenance and chronic medical conditions as risk factors for premature death among older heroin addicts. Journal of Psychiatric Practice 15: 227–234. 19461397 10.1097/01.pra.0000351884.83377.e2

[10] Wapf V, Schaub M, Klaeusler B, Boesch L, Stohler R, et al (2008) The barriers to smoking cessation in Swiss methadone and buprenorphine-maintained patients. Harm reduction journal 5: 1–7. 18348722 10.1186/1477-7517-5-10 PMC2276187

[11] Corbin CB, Pangrazi RP, Franks BD (2000) Definitions: Health, Fitness, and Physical Activity. President's Council on Physical Fitness and Sports Research Digest.

[12] Thompson WR, Gordon NF, Pescatello LS (2009) ACSM's guidelines for exercise testing and prescription: Hubsta Ltd. 10.1249/JSR.0b013e31829a68cf 23851406

[13] Weinstock J, Wadeson HK, VanHeest JL (2012) Exercise as an adjunct treatment for opiate agonist treatment: review of the current research and implementation strategies. Substance Abuse 33: 350–360. 22989278 10.1080/08897077.2012.663327 PMC4631114

[14] Ströhle A, Höfler M, Pfister H, Müller AG, Hoyer J, et al (2007) Physical activity and prevalence and incidence of mental disorders in adolescents and young adults. Psychological medicine 37: 1657–1666. 17579930 10.1017/S003329170700089X

[15] Korhonen T, Kujala UM, Rose RJ, Kaprio J (2009) Physical activity in adolescence as a predictor of alcohol and illicit drug use in early adulthood: a longitudinal population-based twin study. Twin Research and Human Genetics 12: 261–268. 19456218 10.1375/twin.12.3.261 PMC2723718

[16] Buchowski MS, Meade NN, Charboneau E, Park S, Dietrich MS, et al (2011) Aerobic exercise training reduces cannabis craving and use in non-treatment seeking cannabis-dependent adults. PLoS One 6: e17465. 21408154 10.1371/journal.pone.0017465 PMC3050879

[17] Brown RA, Abrantes AM, Read JP, Marcus BH, Jakicic J, et al (2010) A pilot study of aerobic exercise as an adjunctive treatment for drug dependence. Mental health and physical activity 3: 27–34. 20582151 10.1016/j.mhpa.2010.03.001 PMC2889694

[18] Sarah EL, Joseph TC, Michael U, Bess HM (2013) Exercise-based smoking cessation interventions among women. Women's Health 9: 69–84. 10.2217/whe.12.63 PMC5718352 23241156

[19] Brown RA, Abrantes AM, Read JP, Marcus BH, Jakicic J, et al (2009) Aerobic Exercise for Alcohol Recovery: Rationale, Program Description, and Preliminary Findings. Behavior modification 33: 220–249. 19091721 10.1177/0145445508329112 PMC2829243

[20] Miladi GH, Rashidy PA, Fathollahi Y (2012) Anxiety profile in morphine-dependent and withdrawn rats: effect of voluntary exercise. Physiology & behavior 105: 195–202. 21871908 10.1016/j.physbeh.2011.08.010

[21] Lynch WJ, Piehl KB, Acosta G, Peterson AB, Hemby SE (2010) Aerobic exercise attenuates reinstatement of cocaine-seeking behavior and associated neuroadaptations in the prefrontal cortex. Biol Psychiatry 68: 774–777. 20692647 10.1016/j.biopsych.2010.06.022 PMC2949528

[22] Sanchez V, Moore C, Brunzell D, Lynch W (2013) Effect of wheel-running during abstinence on subsequent nicotine-seeking in rats. Psychopharmacology 227: 403–411. 23371488 10.1007/s00213-012-2964-x PMC3656970

[23] Thanos PK, Stamos J, Robison LS, Heyman G, Tucci A, et al (2013) Daily treadmill exercise attenuates cocaine cue-induced reinstatement and cocaine induced locomotor response but increases cocaine-primed reinstatement. Behav Brain Res 239: 8–14. 23103403 10.1016/j.bbr.2012.10.035 PMC3596018

[24] Engelmann AJ, Aparicio MB, Kim A, Sobieraj JC, Yuan CJ, et al (2013) Chronic wheel running reduces maladaptive patterns of methamphetamine intake: regulation by attenuation of methamphetamine-induced neuronal nitric oxide synthase. Brain Structure and Function 219: 657–672. 23443965 10.1007/s00429-013-0525-7 PMC3702684

[25] Hashemi Nosrat Abadi T, Vaghef L, Babri S, Mahmood AM, Beirami M (2013) Effects of different exercise protocols on ethanol-induced spatial memory impairment in adult male rats. Alcohol 47: 309–316. 23683528 10.1016/j.alcohol.2013.01.008

[26] Donaghy ME (1997) The investigation of exercise as an adjunct to the treatment and rehabilitation of the problem drinker: University of Glasgow.

[27] Vickers KS, Patten CA, Lewis BA, Clark MM, Ussher M, et al (2009) Feasibility of an exercise counseling intervention for depressed women smokers. Nicotine & tobacco research 11: 985–995. 19541948 10.1093/ntr/ntp101 PMC2711987

[28] Hillman CH, Drobes DJ (2012) Physical Activity and Cognitive Control: Implications for Drug Abuse. Child Development Perspectives 6: 367–373.

[29] Lynch WJ, Peterson AB, Sanchez V, Abel J, Smith MA (2013) Exercise as a novel treatment for drug addiction: A neurobiological and stage-dependent hypothesis. Neuroscience & Biobehavioral Reviews 37: 1622–1644. 23806439 10.1016/j.neubiorev.2013.06.011 PMC3788047

[30] Pareja GH, Sanchis GF, Mayero S (2013) Exercise as an adjuvant intervention in opiate dependence. Substance Abuse 34: 87–88. 23577897 10.1080/08897077.2012.752778

[31] Smith MA, Lynch WJ (2012) Exercise as a potential treatment for drug abuse: evidence from preclinical studies. Frontiers in Psychiatry 2: 1–8. 10.3389/fpsyt.2011.00082 PMC3276339 22347866

[32] Zschucke E, Heinz A, Strohle A (2012) Exercise and physical activity in the therapy of substance use disorders. ScientificWorldJournal 2012: 1–19. 10.1100/2012/901741 PMC3354725 22629222

[33] Khanna S, Greeson JM (2013) A narrative review of yoga and mindfulness as complementary therapies for addiction. Complementary therapies in medicine 21: 244–252. 23642957 10.1016/j.ctim.2013.01.008 PMC3646290

[34] Posadzki P, Choi J, Lee MS, Ernst E (2014) Yoga for addictions: a systematic review of randomised clinical trials. Focus on Alternative and Complementary Therapies 19: 1–8.

[35] Carim TL, Mitchell SH, Oken BS (2013) Mind–body practices: An alternative, drug-free treatment for smoking cessation? A systematic review of the literature. Drug and alcohol dependence 132: 399–410. 23664122 10.1016/j.drugalcdep.2013.04.014 PMC3770754

[36] Ussher M H TA, Faulkner G (2008) Exercise interventions for smoking cessation. Cochrane Database of Systematic Reviews. 10.1002/14651858.CD002295.pub3 18843632

[37] Ussher MH, Taylor A, Faulkner G (2012) Exercise interventions for smoking cessation. Cochrane Database Syst Rev 1: 1–50. 10.1002/14651858.CD002295.pub4 22258948

[38] Haasova M, Warren F, Ussher M, VanRensberg K, Faulkner GC, et al (2013) The acute effects of physical activity on cigarette cravings Systematic review and meta-analysis with individual participant data (IPD). Addiction 108: 26–37. 22861822 10.1111/j.1360-0443.2012.04034.x

[39] Zhuang SM, An SH, Zhao Y (2013) Yoga Effects on Mood and Quality of Life in Chinese Women Undergoing Heroin Detoxification, a Randomized Controlled Trial Nursing Research. 62: 260–268. 10.1097/NNR.0b013e318292379b 23715475

[40] Pattison LP, McIntosh S, Sexton T, Childers SR, Hemby SE (2014) Changes in dopamine transporter binding in nucleus accumbens following chronic self-administration cocaine: Heroin combinations. Synapse. 10.1002/syn.21755 PMC4687011 24916769

[41] Hopkins ME, Davis FC, Vantieghem MR, Whalen PJ, Bucci DJ (2012) Differential effects of acute and regular physical exercise on cognition and affect. Neuroscience 215: 59–68. 22554780 10.1016/j.neuroscience.2012.04.056 PMC3374855

[42] McMorris T, Graydon J (2000) The effect of incremental exercise on cognitive performance. International Journal of Sport Psychology 31: 66–81.

[43] Swain RA, Harris AB, Wiener EC, Dutka MV, Morris HD, et al (2003) Prolonged exercise induces angiogenesis and increases cerebral blood volume in primary motor cortex of the rat. Neuroscience 117: 1037–1046. 12654355 10.1016/s0306-4522(02)00664-4

[44] van Praag H (2008) Neurogenesis and exercise: past and future directions. Neuromolecular medicine 10: 128–140. 18286389 10.1007/s12017-008-8028-z

[45] Moher D, Liberati A, Tetzlaff J, Altman DG (2009) Preferred reporting items for systematic reviews and meta-analyses: the PRISMA statement. Annals of internal medicine 151: 264–269. 19622511 10.7326/0003-4819-151-4-200908180-00135

[46] Verhagen AP, de Vet HC, de Bie RA, Kessels AG, Boers M, et al (1998) The Delphi list: a criteria list for quality assessment of randomized clinical trials for conducting systematic reviews developed by Delphi consensus. Journal of clinical epidemiology 51: 1235–1241. 10086815 10.1016/s0895-4356(98)00131-0

[47] Team RC (2013) R: A Language and Environment for Statistical Computing. Vienna, Austria: R Foundation for Statistical Computing.

[48] Higgins JP, Thompson SG, Deeks JJ, Altman DG (2003) Measuring inconsistency in meta-analyses. BMJ: British Medical Journal 327: 557–560. 12958120 10.1136/bmj.327.7414.557 PMC192859

[49] Higgins J, Thompson SG (2002) Quantifying heterogeneity in a meta-analysis. Statistics in medicine 21: 1539–1558. 12111919 10.1002/sim.1186

[50] Egger M, Smith GD, Schneider M, Minder C (1997) Bias in meta-analysis detected by a simple, graphical test. Bmj 315: 629–634. 9310563 10.1136/bmj.315.7109.629 PMC2127453

[51] Li M, Chen K, Mo Z (2002) Use of qigong therapy in the detoxification of heroin addicts. Alternative Therapies in Health and Medicine 8: 50–59. 11795622

[52] Huang H, Yang F, Yang SS, Xiao DS, Nie AH, et al (2000) Influence of aerobic training on recovery of herion sddicts. Chinese Journal of Physical Therapy 23: 267–270.

[53] Huang H, Yang F (2000) Exercise therapy adjuvant treatment of herion dependence on clinical observation. Chinese Journal of Drug Abuse Prevention and Treatment 26: 30–31.

[54] Li DX, Zhuang XY, Zhang YP, Guo H, Wang Z, et al (2013) Effects of Tai Chi on the Protracted Abstinence Syndrome: A Time Trial Analysis. The American journal of Chinese medicine 41: 43–57. 23336506 10.1142/S0192415X13500043

[55] Marcus BH, Albrecht AE, Niaura RS, Taylor ER, Simkin LR, et al (1995) Exercise enhances the maintenance of smoking cessation in women. Addictive Behaviors 20: 87–92. 7785485 10.1016/0306-4603(94)00048-4

[56] Marcus BH, Albrecht AE, King TK, Parisi AF, Pinto BM, et al (1999) The efficacy of exercise as an aid for smoking cessation in women: a randomized controlled trial. Archives of Internal Medicine 159: 1229–1235. 10371231 10.1001/archinte.159.11.1229

[57] Ussher M, West R, McEwen A, Taylor A, Steptoe A (2003) Efficacy of exercise counselling as an aid for smoking cessation: a randomized controlled trial. Addiction 98: 523–532. 12653822 10.1046/j.1360-0443.2003.00346.x

[58] Marcus BH, Lewis BA, Hogan J, King TK, Albrecht AE, et al (2005) The efficacy of moderate-intensity exercise as an aid for smoking cessation in women: a randomized controlled trial. Nicotine Tob Res 7: 871–880. 16298722 10.1080/14622200500266056

[59] Prapavessis H, Cameron L, Baldi JC, Robinson S, Borrie K, et al (2007) The effects of exercise and nicotine replacement therapy on smoking rates in women. Addict Behav 32: 1416–1432. 17097814 10.1016/j.addbeh.2006.10.005

[60] Ussher M, West R, McEwen A, Taylor A, Steptoe A (2007) Randomized controlled trial of physical activity counseling as an aid to smoking cessation: 12 month follow-up. Addict Behav 32: 3060–3064. 17499444 10.1016/j.addbeh.2007.04.009

[61] Kinnunen T, Leeman RF, Korhonen T, Quiles ZN, Terwal DM, et al (2008) Exercise as an adjunct to nicotine gum in treating tobacco dependence among women. Nicotine & Tobacco Research 10: 689–703. 18418791 10.1080/14622200801979043 PMC3695732

[62] Williams DM, Whiteley JA, Dunsiger S, Jennings EG, Albrecht AE, et al (2010) Moderate intensity exercise as an adjunct to standard smoking cessation treatment for women: a pilot study. Psychology of Addictive Behaviors 24: 349–354. 20565161 10.1037/a0018332 PMC4075011

[63] Bock BC, Fava JL, Gaskins R, Morrow KM, Williams DM, et al (2012) Yoga as a complementary treatment for smoking cessation in women. J Womens Health (Larchmt) 21: 240–248. 21992583 10.1089/jwh.2011.2963 PMC3304243

[64] Whiteley JA, Williams DM, Dunsiger S, Jennings EG, Ciccolo JT, et al (2012) YMCA commit to quit: randomized trial outcomes. Am J Prev Med 43: 256–262. 22898118 10.1016/j.amepre.2012.05.025

[65] Vedamurthachar A, Janakiramaiah N, Hegde JM, Shetty TK, Subbakrishna D, et al (2006) Antidepressant efficacy and hormonal effects of Sudarshana Kriya Yoga (SKY) in alcohol dependent individuals. Journal of affective disorders 94: 249–253. 16740317 10.1016/j.jad.2006.04.025

[66] Sareen S, Kumari V, Gajebasia KS, Gajebasia NK (2007) Yoga: a tool for improving the quality of life in chronic pancreatitis. World Journal of Gastroenterology 13: 391. 17230607 10.3748/wjg.v13.i3.391 PMC4065893

[67] Burling TA, Seidner AL, Robbins-Sisco D, Krinsky A, Hanser SB (1992) Batter up! Relapse prevention for homeless veteran substance abusers via softball team participation. Journal of Substance Abuse 4: 407–413. 1338187 10.1016/0899-3289(92)90047-2

[68] Martin JE, Calfas KJ, Patten CA, Polarek M, Hofstetter CR, et al (1997) Prospective evaluation of three smoking interventions in 205 recovering alcoholics: One-year results of Project SCRAP-Tobacco. Journal of Consulting and Clinical Psychology 65: 190–194. 9103749 10.1037//0022-006x.65.1.190

[69] Smelson D, Chen KW, Ziedonis D, Andes K, Lennox A, et al (2013) A Pilot Study of Qigong for Reducing Cocaine Craving Early in Recovery. The Journal of Alternative and Complementary Medicine 19: 97–101. 22757968 10.1089/acm.2012.0052 PMC3576894

[70] Roberts V, Maddison R, Simpson C, Bullen C, Prapavessis H (2012) The acute effects of exercise on cigarette cravings, withdrawal symptoms, affect, and smoking behaviour: systematic review update and meta-analysis. Psychopharmacology (Berl) 222: 1–15. 22585034 10.1007/s00213-012-2731-z

[71] Sarkar DK, Sengupta A, Zhang C, Boyadjieva N, Murugan S (2012) Opiate antagonist prevents µ-and δ-opiate receptor dimerization to facilitate ability of agonist to control ethanol-altered natural killer cell functions and mammary tumor growth. Journal of Biological Chemistry 287: 16734–16747. 22451667 10.1074/jbc.M112.347583 PMC3351352

[72] Silveri MM (2014) GABAergic contributions to alcohol responsivity during adolescence: Insights from preclinical and clinical studies. Pharmacology & Therapeutics 143: 197–216. 24631274 10.1016/j.pharmthera.2014.03.001 PMC4454465

[73] Gilman JM, Ramchandani VA, Davis MB, Bjork JM, Hommer DW (2008) Why we like to drink: a functional magnetic resonance imaging study of the rewarding and anxiolytic effects of alcohol. The Journal of Neuroscience 28: 4583–4591. 18448634 10.1523/JNEUROSCI.0086-08.2008 PMC2730732

[74] Berrettini W (2013) Opioid pharmacogenetics of alcohol addiction. Alcohol: Science, Policy and Public Health 3: 97–113. 10.1101/cshperspect.a012203 PMC3685876 23729643

[75] Dajas-Bailador F, Wonnacott S (2004) Nicotinic acetylcholine receptors and the regulation of neuronal signalling. Trends in pharmacological sciences 25: 317–324. 15165747 10.1016/j.tips.2004.04.006

[76] Coe JW, Brooks PR, Vetelino MG, Wirtz MC, Arnold EP, et al (2005) Varenicline: an α4β2 nicotinic receptor partial agonist for smoking cessation. Journal of medicinal chemistry 48: 3474–3477. 15887955 10.1021/jm050069n

[77] Sala M, Braida D, Pucci L, Manfredi I, Marks MJ, et al (2013) CC4, a dimer of cytisine, is a selective partial agonist at α4β2/α6β2 nAChR with improved selectivity for tobacco smoking cessation. British journal of pharmacology 168: 835–849. 22957729 10.1111/j.1476-5381.2012.02204.x PMC3631374

[78] Roessler KK, Bilberg R, Jensen K, Kjaergaard A-S, Dervisevic A, et al (2013) Exercise as Treatment for Alcohol Dependence. Sport Science Review 22: 205–216.

[79] Taylor AH, Oh H, Cullen S (2013) Acute effect of exercise on alcohol urges and attentional bias towards alcohol related images in high alcohol consumers. Mental Health and Physical Activity 6: 220–226.

[80] Karoly HC, Stevens CJ, Thayer RE, Magnan RE, Bryan AD, et al (2013) Aerobic Exercise Moderates the Effect of Heavy Alcohol Consumption on White Matter Damage. Alcoholism: Clinical and Experimental Research 37: 1508–1515. 10.1111/acer.12135 PMC3708994 23551199

[81] Koo JW, Mazei-Robison MS, Chaudhury D, Juarez B, LaPlant Q, et al (2012) BDNF is a negative modulator of morphine action. science 338: 124–128. 23042896 10.1126/science.1222265 PMC3547365

[82] Gomez PF, Hillman C (2013) The Influence of Exercise on Cognitive Abilities. Comprehensive Physiology 3: 403–428. 23720292 10.1002/cphy.c110063 PMC3951958

[83] Coelho FG, Gobbi S, Andreatto CA, Corazza DI, Pedroso RV, et al (2013) Physical exercise modulates peripheral levels of brain-derived neurotrophic factor (BDNF): a systematic review of experimental studies in the elderly. Arch Gerontol Geriatr 56: 10–15. 22749404 10.1016/j.archger.2012.06.003

[84] Thomas AG, Dennis A, Bandettini PA, Johansen-Berg H (2012) The effects of aerobic activity on brain structure. Frontiers in psychology 3: 1–9. 22470361 10.3389/fpsyg.2012.00086 PMC3311131

[85] Lin TW, Kuo YM (2013) Exercise Benefits Brain Function: The Monoamine Connection. Brain Sciences 3: 39–53. 24961306 10.3390/brainsci3010039 PMC4061837

[86] Lan C, Lai JS, Chen SY, Wong MK (1998) 12-month Tai Chi training in the elderly: its effect on health fitness. Medicine and Science in Sports and Exercise 30: 345–351. 9526879 10.1097/00005768-199803000-00003

[87] Ahn S, Song R (2012) Effects of Tai Chi Exercise on glucose control, neuropathy scores, balance, and quality of life in patients with type 2 diabetes and neuropathy. The Journal of Alternative and Complementary Medicine 18: 1172–1178. 22985218 10.1089/acm.2011.0690 PMC3513979

[88] Wu Y, Wang Y, Burgess EO, Wu J (2013) The effects of Tai Chi exercise on cognitive function in older adults: A meta-analysis. Journal of Sport and Health Science 2: 193–203.

[89] Pan L, Yan J, Guo Y, Yan J (2013) Effects of Tai Chi training on exercise capacity and quality of life in patients with chronic heart failure: a meta-analysis. European journal of heart failure 15: 316–323. 23099355 10.1093/eurjhf/hfs170

[90] Kjos V, Etnier JL (2006) Pilot study comparing physical and psychological responses in medical qigong and walking. Journal of aging and physical activity 14: 241–253. 17090803 10.1123/japa.14.3.241

[91] DiPietro L, Seeman TE, Stachenfeld NS, Katz LD, Nadel ER (1998) Moderate-intensity aerobic training improves glucose tolerance in aging independent of abdominal adiposity. Journal of the American Geriatrics Society 46: 875–879. 9670875 10.1111/j.1532-5415.1998.tb02722.x

[92] Ward S, McCluney N, Bosch P (2013) Heart Rate Response to Vinyasa Yoga in Healthy Adults. J Yoga Phys Ther 3: 1–15.

[93] Khalsa SBS, Khalsa GS, Khalsa HK, Khalsa MK (2008) Evaluation of a residential Kundalini yoga lifestyle pilot program for addiction in India. Journal of ethnicity in substance abuse 7: 67–79. 19842301 10.1080/15332640802081968

[94] Elibero A, Janse Van Rensburg K, Drobes DJ (2011) Acute effects of aerobic exercise and Hatha yoga on craving to smoke. Nicotine Tob Res 13: 1140–1148. 21849414 10.1093/ntr/ntr163

[95] Kinnunen TH, Korhonen T, Craft LL, Perna FM (2010) Treating tobacco dependence in women with exercise: Review on effectiveness and mechanisms. International Journal of Sport and Exercise Psychology 8: 48–60.

